# 7-Chloro-5-(2-ethoxy­phen­yl)-1-methyl-3-propyl-2,6-dihydro-1*H*-pyrazolo[4,3-*d*]pyrimidine

**DOI:** 10.1107/S1600536809033911

**Published:** 2009-09-05

**Authors:** Ming-Qiu Zhou, Kai Zhu, Xiao-Ping Lv, Ping-Fang Han, Ping Wei

**Affiliations:** a College of Biotechnology and Pharmaceutical Engineering, Nanjing University of Technolgy, Xinmofan Road No. 5 Nanjing, Nanjing 210009, People’s Republic of China

## Abstract

In the title compound, C_17_H_21_ClN_4_O, the benzene ring is oriented at dihedral angles of 1.59 (3) and 1.27 (3)° with respect to the pyrimidine and pyrazole rings, while the dihedral angle between the pyrimidine and pyrazole rings is 0.83 (3)°. An intra­molecular N—H⋯O hydrogen bond results in the formation of a planar (r.m.s. deviation 0.004 Å) six-membered ring.

## Related literature

For a related structure, see: Rajesh & Joshi (2007 ▶). For bond-length data, see: Allen *et al.* (1987 ▶).
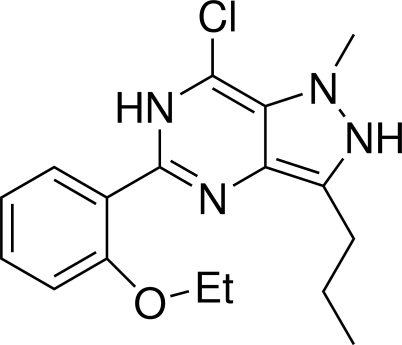


## Experimental

### 

#### Crystal data

C_17_H_21_ClN_4_O
*M* *_r_* = 332.83
Triclinic, 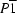

*a* = 4.6700 (9) Å
*b* = 11.647 (2) Å
*c* = 16.064 (3) Å
α = 78.56 (3)°
β = 86.75 (3)°
γ = 79.81 (3)°
*V* = 842.7 (3) Å^3^
*Z* = 2
Mo *K*α radiation
μ = 0.24 mm^−1^
*T* = 294 K
0.30 × 0.10 × 0.10 mm

#### Data collection

Enraf–Nonius CAD-4 diffractometer
Absorption correction: ψ scan (North *et al.*, 1968 ▶) *T*_min_ = 0.932, *T*_max_ = 0.977
3470 measured reflections
3061 independent reflections
2353 reflections with *I* > 2σ(*I*)
*R*_int_ = 0.033
3 standard reflections frequency: 120 min intensity decay: 1%

#### Refinement

*R*[*F* ^2^ > 2σ(*F* ^2^)] = 0.064
*wR*(*F* ^2^) = 0.182
*S* = 1.00
3061 reflections
208 parameters
H-atom parameters constrained
Δρ_max_ = 0.38 e Å^−3^
Δρ_min_ = −0.52 e Å^−3^

### 

Data collection: *CAD-4 Software* (Enraf–Nonius, 1989 ▶); cell refinement: *CAD-4 Software*; data reduction: *XCAD4* (Harms & Wocadlo, 1995 ▶); program(s) used to solve structure: *SHELXS97* (Sheldrick, 2008 ▶); program(s) used to refine structure: *SHELXL97* (Sheldrick, 2008 ▶); molecular graphics: *ORTEP-3 for Windows* (Farrugia, 1997 ▶) and *PLATON* (Spek, 2009 ▶); software used to prepare material for publication: *SHELXL97* and *PLATON*.

## Supplementary Material

Crystal structure: contains datablocks global, I. DOI: 10.1107/S1600536809033911/hk2761sup1.cif

Structure factors: contains datablocks I. DOI: 10.1107/S1600536809033911/hk2761Isup2.hkl

Additional supplementary materials:  crystallographic information; 3D view; checkCIF report

## Figures and Tables

**Table 1:** Hydrogen-bond geometry (Å, °)

*D*—H⋯*A*	*D*—H	H⋯*A*	*D*⋯*A*	*D*—H⋯*A*
N1—H1*A*⋯O1	0.86	1.91	2.616 (3)	138

**Fig. 1.**
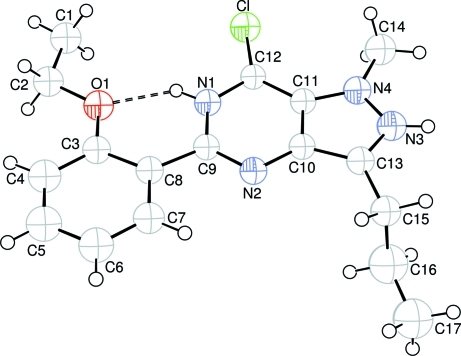
The molecular structure of the title molecule, with the atom-numbering scheme. Hydrogen bond is shown as dashed line.

## supplementary crystallographic information

### Comment

Some derivatives of 5-(2-ethoxyphenyl)-1-methyl-3-propyl-1,6-dihydro-7H -pyrazolo[4,3-*d*]pyrimidin-7-one are important chemical materials. We report herein the crystal structure of the title compound.

In the molecule of the title compound, (Fig. 1), the bond lengths (Allen *et al.*,  1987) and angles are within normal ranges. Rings A (C3-C8), B (N1/N2/C9-C12) and C (N3/N4/C10/C11/C13) are, of course, planar. The dihedral angles between them are A/B = 1.59 (3), A/C = 1.27 (3) and B/C = 0.83 (3) °. The intramolecular N-H···O hydrogen bond (Table 1) results in the formation of a planar six-membered ring D (O1/N1/C3/C8/C9/H1A), which is oriented with respect to the other rings at dihedral angles of A/D = 1.01 (3), B/D = 0.63 (3) and C/D = 0.83 (3) °. So, the rings are almost coplanar.

### Experimental

For the preparation of the title compound, 5-(2-ethoxyphenyl)-1-methyl-3-propyl -1,6-dihydro-7H-pyrazolo[4,3-*d*]pyrimidin-7-one (15.6 g) and phosphorus trichloride (13.7 g) were added into carbon tetrachloride (100 ml) at 345-350 K. The gross products were extracted with n-hexane, dried under vaccum, and then recrystallized in dichloromethane. Finally the title compound is obtained (yield; 1.5 g) (Rajesh *et al.*, 2007). Crystals suitable for X-ray analysis were obtained by slow evaporation of a methanol solution.

### Refinement

H atoms were positioned geometrically with N-H = 0.86 Å (for NH) and C-H = 0.93, 0.97 and 0.96 Å for aromatic, methylene and methyl H atoms, respectively, and constrained to ride on their parent atoms with U_iso_(H) = xU_eq_(C,N), where x = 1.5 for methyl H and x = 1.2 for all other H atoms.

### Crystal data

**Table:**

C_17_H_21_ClN_4_O	*Z* = 2
*M**_r_* = 332.83	*F*(000) = 352
Triclinic, *P*1	*D*_x_ = 1.312 Mg m^−^^3^
Hall symbol: -P 1	Mo *K*α radiation, λ = 0.71073 Å
*a* = 4.6700 (9) Å	Cell parameters from 25 reflections
*b* = 11.647 (2) Å	θ = 9–13°
*c* = 16.064 (3) Å	µ = 0.24 mm^−^^1^
α = 78.56 (3)°	*T* = 294 K
β = 86.75 (3)°	BlocK, yellow
γ = 79.81 (3)°	0.30 × 0.10 × 0.10 mm
*V* = 842.7 (3) Å^3^	

### Data collection

**Table:**

Enraf–Nonius CAD-4 diffractometer	2353 reflections with *I* > 2σ(*I*)
Radiation source: fine-focus sealed tube	*R*_int_ = 0.033
graphite	θ_max_ = 25.3°, θ_min_ = 1.3°
ω/2θ scans	*h* = 0→5
Absorption correction: ψ scan (North *et al.*, 1968)	*k* = −13→13
*T*_min_ = 0.932, *T*_max_ = 0.977	*l* = −19→19
3470 measured reflections	3 standard reflections every 120 min
3061 independent reflections	intensity decay: 1%

### Refinement

**Table:**

Refinement on *F*^2^	Primary atom site location: structure-invariant direct methods
Least-squares matrix: full	Secondary atom site location: difference Fourier map
*R*[*F*^2^ > 2σ(*F*^2^)] = 0.064	Hydrogen site location: inferred from neighbouring sites
*wR*(*F*^2^) = 0.182	H-atom parameters constrained
*S* = 1.00	*w* = 1/[σ^2^(*F*_o_^2^) + (0.1*P*)^2^ + 0.6*P*] where *P* = (*F*_o_^2^ + 2*F*_c_^2^)/3
3061 reflections	(Δ/σ)_max_ = 0.001
208 parameters	Δρ_max_ = 0.38 e Å^−^^3^
0 restraints	Δρ_min_ = −0.52 e Å^−^^3^

### Special details

**Table:**

Geometry. All e.s.d.'s (except the e.s.d. in the dihedral angle between two l.s. planes) are estimated using the full covariance matrix. The cell e.s.d.'s are taken into account individually in the estimation of e.s.d.'s in distances, angles and torsion angles; correlations between e.s.d.'s in cell parameters are only used when they are defined by crystal symmetry. An approximate (isotropic) treatment of cell e.s.d.'s is used for estimating e.s.d.'s involving l.s. planes.
Refinement. Refinement of *F*^2^ against ALL reflections. The weighted *R*-factor *wR* and goodness of fit *S* are based on *F*^2^, conventional *R*-factors *R* are based on *F*, with *F* set to zero for negative *F*^2^. The threshold expression of *F*^2^ > σ(*F*^2^) is used only for calculating *R*-factors(gt) *etc*. and is not relevant to the choice of reflections for refinement. *R*-factors based on *F*^2^ are statistically about twice as large as those based on *F*, and *R*- factors based on ALL data will be even larger.

### Fractional atomic coordinates and isotropic or equivalent isotropic displacement parameters (Å^2^)

**Table:**

	*x*	*y*	*z*	*U*_iso_*/*U*_eq_	
Cl	0.45734 (19)	0.18716 (7)	0.56202 (5)	0.0581 (3)	
O1	0.4864 (5)	0.49028 (18)	0.68108 (14)	0.0528 (6)	
N1	0.6871 (5)	0.2658 (2)	0.68131 (14)	0.0420 (6)	
H1A	0.5753	0.3313	0.6613	0.050*	
N2	1.0314 (5)	0.1732 (2)	0.78486 (14)	0.0414 (6)	
N3	1.1345 (6)	−0.1091 (2)	0.72458 (16)	0.0506 (7)	
H3A	1.2075	−0.1828	0.7269	0.061*	
N4	0.9354 (6)	−0.0435 (2)	0.66884 (16)	0.0466 (6)	
C1	0.1508 (7)	0.5709 (3)	0.5722 (2)	0.0605 (9)	
H1B	0.0284	0.6416	0.5443	0.091*	
H1C	0.0335	0.5119	0.5956	0.091*	
H1D	0.2893	0.5410	0.5319	0.091*	
C2	0.3079 (7)	0.5992 (3)	0.6417 (2)	0.0550 (8)	
H2B	0.4278	0.6587	0.6189	0.066*	
H2C	0.1703	0.6297	0.6829	0.066*	
C3	0.6554 (7)	0.4882 (3)	0.74759 (19)	0.0452 (7)	
C4	0.6545 (8)	0.5877 (3)	0.7839 (2)	0.0597 (9)	
H4A	0.5325	0.6587	0.7632	0.072*	
C5	0.8344 (9)	0.5806 (3)	0.8503 (2)	0.0671 (10)	
H5A	0.8325	0.6470	0.8744	0.081*	
C6	1.0161 (9)	0.4770 (3)	0.8812 (2)	0.0635 (9)	
H6A	1.1384	0.4735	0.9257	0.076*	
C7	1.0177 (7)	0.3782 (3)	0.8465 (2)	0.0528 (8)	
H7A	1.1418	0.3082	0.8683	0.063*	
C8	0.8384 (6)	0.3800 (3)	0.77958 (18)	0.0428 (7)	
C9	0.8566 (6)	0.2674 (2)	0.74811 (17)	0.0393 (6)	
C10	1.0325 (6)	0.0743 (2)	0.75138 (17)	0.0397 (6)	
C11	0.8671 (6)	0.0690 (2)	0.68347 (17)	0.0398 (6)	
C12	0.6759 (6)	0.1717 (2)	0.64296 (17)	0.0389 (6)	
C13	1.1973 (6)	−0.0409 (3)	0.77464 (19)	0.0443 (7)	
C14	0.8296 (8)	−0.0978 (3)	0.6049 (2)	0.0606 (9)	
H14A	0.9214	−0.1795	0.6116	0.091*	
H14B	0.8752	−0.0562	0.5493	0.091*	
H14C	0.6226	−0.0934	0.6116	0.091*	
C15	1.3994 (7)	−0.0863 (3)	0.84708 (19)	0.0502 (8)	
H15A	1.4993	−0.0232	0.8551	0.060*	
H15B	1.5449	−0.1506	0.8332	0.060*	
C16	1.2456 (9)	−0.1304 (5)	0.9283 (2)	0.0868 (14)	
H16A	1.0974	−0.0661	0.9409	0.104*	
H16B	1.1471	−0.1936	0.9197	0.104*	
C17	1.4345 (10)	−0.1757 (5)	1.0040 (2)	0.0865 (13)	
H17A	1.3173	−0.2021	1.0527	0.130*	
H17B	1.5283	−0.1132	1.0147	0.130*	
H17C	1.5793	−0.2410	0.9932	0.130*	

### Atomic displacement parameters (Å^2^)

**Table:**

	*U*^11^	*U*^22^	*U*^33^	*U*^12^	*U*^13^	*U*^23^
Cl	0.0624 (5)	0.0586 (5)	0.0533 (5)	−0.0064 (4)	−0.0254 (4)	−0.0076 (4)
O1	0.0595 (13)	0.0387 (11)	0.0578 (13)	0.0041 (10)	−0.0213 (11)	−0.0087 (9)
N1	0.0438 (13)	0.0384 (13)	0.0421 (13)	−0.0020 (10)	−0.0136 (11)	−0.0050 (10)
N2	0.0417 (13)	0.0432 (13)	0.0385 (12)	−0.0065 (10)	−0.0090 (10)	−0.0040 (10)
N3	0.0554 (16)	0.0390 (13)	0.0539 (15)	0.0040 (12)	−0.0135 (12)	−0.0079 (11)
N4	0.0530 (15)	0.0395 (13)	0.0472 (14)	−0.0013 (11)	−0.0119 (12)	−0.0106 (11)
C1	0.0518 (19)	0.061 (2)	0.063 (2)	0.0071 (16)	−0.0128 (16)	−0.0078 (17)
C2	0.0521 (19)	0.0433 (17)	0.063 (2)	0.0070 (14)	−0.0095 (16)	−0.0055 (15)
C3	0.0453 (16)	0.0440 (16)	0.0469 (17)	−0.0074 (13)	−0.0020 (13)	−0.0102 (13)
C4	0.065 (2)	0.0452 (18)	0.070 (2)	−0.0044 (16)	−0.0089 (18)	−0.0163 (16)
C5	0.081 (3)	0.057 (2)	0.072 (2)	−0.0113 (19)	−0.012 (2)	−0.0298 (18)
C6	0.074 (2)	0.065 (2)	0.057 (2)	−0.0163 (19)	−0.0199 (18)	−0.0194 (17)
C7	0.0583 (19)	0.0499 (18)	0.0516 (18)	−0.0076 (15)	−0.0168 (15)	−0.0102 (14)
C8	0.0430 (16)	0.0428 (16)	0.0432 (15)	−0.0088 (13)	−0.0052 (13)	−0.0068 (12)
C9	0.0376 (14)	0.0407 (15)	0.0383 (14)	−0.0069 (12)	−0.0037 (12)	−0.0037 (12)
C10	0.0399 (15)	0.0404 (15)	0.0374 (14)	−0.0049 (12)	−0.0043 (12)	−0.0044 (12)
C11	0.0409 (15)	0.0400 (15)	0.0379 (14)	−0.0066 (12)	−0.0045 (12)	−0.0048 (12)
C12	0.0376 (14)	0.0413 (15)	0.0377 (14)	−0.0097 (12)	−0.0031 (12)	−0.0040 (12)
C13	0.0419 (16)	0.0431 (16)	0.0441 (16)	−0.0004 (12)	−0.0055 (13)	−0.0042 (13)
C14	0.074 (2)	0.0503 (19)	0.061 (2)	−0.0034 (17)	−0.0181 (18)	−0.0203 (16)
C15	0.0476 (17)	0.0463 (17)	0.0524 (18)	0.0036 (13)	−0.0128 (14)	−0.0060 (14)
C16	0.060 (2)	0.135 (4)	0.057 (2)	−0.028 (2)	−0.0162 (19)	0.014 (2)
C17	0.081 (3)	0.117 (4)	0.055 (2)	−0.024 (3)	−0.015 (2)	0.010 (2)

### Geometric parameters (Å, °)

**Table:**

Cl—C12	1.660 (3)	C5—C6	1.367 (5)
O1—C2	1.439 (4)	C5—H5A	0.9300
O1—C3	1.358 (4)	C6—C7	1.373 (5)
N1—C9	1.374 (3)	C6—H6A	0.9300
N1—C12	1.369 (4)	C7—C8	1.395 (4)
N1—H1A	0.8600	C7—H7A	0.9300
N2—C9	1.304 (4)	C8—C9	1.484 (4)
N2—C10	1.363 (4)	C10—C11	1.389 (4)
N3—N4	1.350 (3)	C10—C13	1.414 (4)
N3—C13	1.315 (4)	C11—C12	1.425 (4)
N3—H3A	0.8600	C13—C15	1.493 (4)
N4—C11	1.356 (4)	C14—H14A	0.9600
N4—C14	1.458 (4)	C14—H14B	0.9600
C1—C2	1.489 (5)	C14—H14C	0.9600
C1—H1B	0.9600	C15—C16	1.497 (5)
C1—H1C	0.9600	C15—H15A	0.9700
C1—H1D	0.9600	C15—H15B	0.9700
C2—H2B	0.9700	C16—C17	1.496 (5)
C2—H2C	0.9700	C16—H16A	0.9700
C3—C4	1.396 (4)	C16—H16B	0.9700
C3—C8	1.412 (4)	C17—H17A	0.9600
C4—C5	1.375 (5)	C17—H17B	0.9600
C4—H4A	0.9300	C17—H17C	0.9600
			
C3—O1—C2	120.4 (2)	C3—C8—C9	125.6 (3)
C12—N1—C9	127.4 (2)	N2—C9—N1	122.1 (3)
C12—N1—H1A	116.3	N2—C9—C8	119.1 (3)
C9—N1—H1A	116.3	N1—C9—C8	118.8 (2)
C9—N2—C10	114.6 (2)	N2—C10—C11	125.1 (3)
C13—N3—N4	108.1 (2)	N2—C10—C13	129.4 (3)
C13—N3—H3A	125.9	C11—C10—C13	105.4 (3)
N4—N3—H3A	125.9	N4—C11—C10	106.8 (2)
N3—N4—C11	110.3 (2)	N4—C11—C12	132.7 (3)
N3—N4—C14	119.5 (2)	C10—C11—C12	120.5 (3)
C11—N4—C14	130.2 (3)	N1—C12—C11	110.1 (2)
C2—C1—H1B	109.5	N1—C12—Cl	120.5 (2)
C2—C1—H1C	109.5	C11—C12—Cl	129.4 (2)
H1B—C1—H1C	109.5	N3—C13—C10	109.3 (3)
C2—C1—H1D	109.5	N3—C13—C15	122.6 (3)
H1B—C1—H1D	109.5	C10—C13—C15	127.9 (3)
H1C—C1—H1D	109.5	N4—C14—H14A	109.5
O1—C2—C1	106.9 (3)	N4—C14—H14B	109.5
O1—C2—H2B	110.3	H14A—C14—H14B	109.5
C1—C2—H2B	110.3	N4—C14—H14C	109.5
O1—C2—H2C	110.3	H14A—C14—H14C	109.5
C1—C2—H2C	110.3	H14B—C14—H14C	109.5
H2B—C2—H2C	108.6	C13—C15—C16	112.7 (3)
O1—C3—C4	122.6 (3)	C13—C15—H15A	109.1
O1—C3—C8	117.3 (3)	C16—C15—H15A	109.1
C4—C3—C8	120.1 (3)	C13—C15—H15B	109.1
C5—C4—C3	119.9 (3)	C16—C15—H15B	109.1
C5—C4—H4A	120.0	H15A—C15—H15B	107.8
C3—C4—H4A	120.0	C17—C16—C15	115.6 (3)
C6—C5—C4	120.8 (3)	C17—C16—H16A	108.4
C6—C5—H5A	119.6	C15—C16—H16A	108.4
C4—C5—H5A	119.6	C17—C16—H16B	108.4
C5—C6—C7	119.9 (3)	C15—C16—H16B	108.4
C5—C6—H6A	120.1	H16A—C16—H16B	107.5
C7—C6—H6A	120.1	C16—C17—H17A	109.5
C6—C7—C8	121.8 (3)	C16—C17—H17B	109.5
C6—C7—H7A	119.1	H17A—C17—H17B	109.5
C8—C7—H7A	119.1	C16—C17—H17C	109.5
C7—C8—C3	117.5 (3)	H17A—C17—H17C	109.5
C7—C8—C9	116.9 (3)	H17B—C17—H17C	109.5
			
C3—O1—C2—C1	−179.6 (3)	C6—C7—C8—C9	−179.3 (3)
C2—O1—C3—C4	3.4 (5)	O1—C3—C8—C7	178.4 (3)
C2—O1—C3—C8	−176.2 (3)	C4—C3—C8—C7	−1.2 (5)
C12—N1—C9—N2	−1.2 (5)	O1—C3—C8—C9	−1.6 (5)
C12—N1—C9—C8	179.2 (3)	C4—C3—C8—C9	178.8 (3)
C9—N1—C12—C11	1.1 (4)	C7—C8—C9—N2	1.4 (4)
C9—N1—C12—Cl	−179.6 (2)	C3—C8—C9—N2	−178.6 (3)
C10—N2—C9—N1	0.4 (4)	C7—C8—C9—N1	−179.0 (3)
C10—N2—C9—C8	180.0 (2)	C3—C8—C9—N1	1.1 (4)
C9—N2—C10—C11	0.3 (4)	N2—C10—C11—N4	178.7 (3)
C9—N2—C10—C13	178.9 (3)	C13—C10—C11—N4	−0.2 (3)
C13—N3—N4—C11	−0.4 (3)	N2—C10—C11—C12	−0.3 (4)
C13—N3—N4—C14	179.3 (3)	C13—C10—C11—C12	−179.1 (3)
N4—N3—C13—C10	0.2 (3)	N4—C11—C12—N1	−179.1 (3)
N4—N3—C13—C15	176.7 (3)	C10—C11—C12—N1	−0.4 (4)
N3—N4—C11—C10	0.3 (3)	N4—C11—C12—Cl	1.7 (5)
C14—N4—C11—C10	−179.3 (3)	C10—C11—C12—Cl	−179.6 (2)
N3—N4—C11—C12	179.1 (3)	N2—C10—C13—N3	−178.9 (3)
C14—N4—C11—C12	−0.5 (6)	C11—C10—C13—N3	−0.1 (3)
O1—C3—C4—C5	−178.9 (3)	N2—C10—C13—C15	4.9 (5)
C8—C3—C4—C5	0.7 (5)	C11—C10—C13—C15	−176.3 (3)
C3—C4—C5—C6	0.3 (6)	N3—C13—C15—C16	−90.3 (4)
C4—C5—C6—C7	−0.7 (6)	C10—C13—C15—C16	85.5 (4)
C5—C6—C7—C8	0.2 (6)	C13—C15—C16—C17	−179.2 (4)
C6—C7—C8—C3	0.7 (5)		

### Hydrogen-bond geometry (Å, °)

**Table:**

*D*—H···*A*	*D*—H	H···*A*	*D*···*A*	*D*—H···*A*
N1—H1A···O1	0.86	1.91	2.616 (3)	138
